# Blood Derivatives in the Therapy of Ocular Surface Diseases

**DOI:** 10.3390/ijms262211097

**Published:** 2025-11-17

**Authors:** Piotr Stępień, Tomasz Żarnowski, Dominika Wróbel-Dudzińska

**Affiliations:** Department of Diagnostic and Microsurgery of Glaucoma, Medical University of Lublin, 20-059 Lublin, Poland; piotr.stepien.dysk@gmail.com (P.S.); zarnowskit@poczta.onet.pl (T.Ż.)

**Keywords:** autologous serum, blood derivatives, platelet-rich plasma, platelet rich in growth factor, platelet lysate, umbilical cord, ocular surface

## Abstract

The ocular surface is a structure crucial to maintaining eye health and proper vision. Unfortunately, ocular surface diseases functioning as chronic epithelial defects, inflammation, impaired healing, require immediate regenerative repair treatment that can restore tissue integrity and function. Conventional therapies, such as artificial tears and topical anti-inflammatory agents, principally provide symptomatic relief without addressing the underlying biological deficits, thus leading to incomplete or delayed recovery. Therefore, blood derivatives have emerged as a promising bioactive therapy that not only lubricates but also actively promotes regeneration through the delivery of cytokines, growth factors, and vitamins naturally present in blood. Due to their properties mimicking the components of natural tears, autologous origin, biocompatibility and capacity to enhance tissue repair, they have emerged as a cornerstone in regenerative medicine. Therefore, the purpose of this review was to compare the evolution, positive aspects, and drawbacks, in order to demonstrate the molecular mechanism of action and the therapeutic efficacy of different blood derivates at treating ocular surface disease. Over time, these biologic preparations have evolved from the use of simple traditional serum-based derivatives to more advanced platelet-rich products, underscoring the evolving understanding of platelet-driven molecular and cellular mechanisms in tissue regeneration. Despite their widespread use, we would like to highlight the current limitations related to the lack of standardized preparation protocols, variability in composition, and evidence-based integration into clinical practice. Finally, this review highlights contemporary research trends and depicts future directions advancing the field. Key priorities include the establishment of standardized, reproducible preparation protocols; the development of next-generation platelet-derived concentrates and biomaterials; and the integration of multi-omics technologies to achieve comprehensive profiling of their biological and therapeutic activity. Moving toward methodological standardization and the execution of well-designed, high-quality comparative clinical trials will be essential to reinforce the scientific foundation, enhance translational potential, and ensure the clinical reliability of blood-derived therapies in modern regenerative medicine.

## 1. Introduction

The ocular surface is a complex and delicate structure that comprises cornea, tear film, conjunctiva, main and accessory lacrimal glands, meibomian gland, and eyelashes with Moll and Zeis glands. All these components are functionally connected by epithelia, innervation, and the endocrine, vascular, and immune systems. Undisturbed, the synergistic function of ocular surface system is crucial for maintaining eye health and visual function, as it ensures the smooth refractive surface of the cornea and serves as the first barrier against environmental insults. However, due to its direct exposure to environmental factors, blinking, and physical abrasions, the ocular surface is subject to desiccation, injury, and pathogens, which can disrupt its function and lead to ocular surface diseases [1].

Tear film, which covers the ocular surface, consists of three main components: the lipid, aqueous, and mucin layers. The aqueous layer, produced by the lacrimal glands, contains the largest fraction of tears. It delivers essential nutrients and oxygen to the surface of the cornea and removes debris and foreign bodies. It consists not only of water and electrolytes, but also numerous growth factors, proteins, and enzymes that are crucial for maintaining ocular surface health. The variety of growth factors present in tears includes the epidermal growth factor (EGF), fibroblast growth factor (FGF), keratinocyte growth factor (KGF), transforming growth factor-beta (TGF-β), nerve growth factor (NGF), hepatocyte growth factor (HGF), vascular endothelial growth factor (VEGF), and insulin-like growth factor (IGF), with functions essential to promoting corneal epithelial cell proliferation, differentiation, wound healing, and maintaining the ocular surface’s protective barrier. EGF and HGF are known to inhibit terminal differentiation, and to promote the proliferation and migration of corneal epithelial cells. TGF-β inhibits proliferation while inducing the normal differentiation of epithelial cells, and plays a role in modulating inflammation and fibrosis. NGF is thought to increase the proliferation and differentiation of corneal epithelial cells and fibroblasts as well as to stimulate nerve regrowth. KGF promotes corneal epithelial cell growth. FGF has a mitogenic effect on corneal epithelial, endothelial, and stromal cells, as well as promoting their migration. IGF stimulates the growth and migration of corneal epithelial cells. Platelet-derived growth factor (PDGF), while it may not be a regular component of tears in the unwounded eye, plays a significant role in corneal wound healing, as it increases the mitosis and migration of corneal fibroblasts and stimulates the chemotaxis of epithelial cells [2,3]. Cornea wound healing is also mediated by fibronectin, a multifunctional glycoprotein which appears at the wound site when the cornea is injured, and enhances the migration of corneal epithelial cells [4] and vitamin A, which supports the differentiation and maintenance of the corneal and conjunctival epithelium [5].

Current standard therapies for ocular surface diseases, such as artificial tears, anti-inflammatory medications, and immunomodulatory agents, often aim to provide symptomatic relief or reduce inflammation. However, when the healing capacity of the eye is impaired, these treatments may not always be sufficient, as they are focused on managing symptoms rather than regenerating eye surface tissues.

## 2. Molecular Basis for Blood Derivatives’ Effectiveness in Treating Ocular Surface Diseases

Blood derivatives have gained rising attention in regenerative medicine due to high content of various biologically active elements such as cells, growth factors, proteins, and cytokines engaged in wound healing process, and their potential to restore the integrity of damaged tissues. Their positive effects on tissue regeneration were shown in several fields of medicine [6].

Blood derivatives owe their efficacy in treating ocular surface diseases to their composition, which is similar to that of natural tears. They provide bioactive ingredients essential for healing, managing inflammation, and epithelial regeneration, such as EGF, NGF, IGF, fibronectin, PDGF, VEGF, vitamin A, substance P, and TGF-β1, which are not present in artificial tears [7,8,9,10]. Moreover, unlike artificial tears, blood-derived eye drops are characterized by natural composition and lack of preservatives or synthetic components which may induce irritation and inflammation of the eye surface [11].

### 2.1. TGF-β

The transforming growth factor-β (TGF-β) family is a group of over 40 polypeptids that play crucial role in modulation of inflammatory responses and tissue repair. In the human cornea TGF-β1, TGF-β2, and TGF-β3 can be found in epithelial cells and keratocytes and take part in corneal wound healing process [12]. The expression of these isoforms changes following injury; the level of TGF-β1 decreases, while TGF-β2 increases and TGF-β3 is not detectable at all. Following injury, TGF-β suppresses the production of proinflammatory cytokines and inhibits the activation and function of neutrophils, while stimulating production of anti-inflammatory molecules, such as interleukin-10 and lipoxins, as well as taking part in the recruitment of regulatory T cells, which prevent excessive immune responses [13]. TGF-β was shown to have two-way functional abilities, as it is able to inhibit various cellular processes and stimulate them depending on the circumstances. It induces extracellular matrix proteins, collagen, and fibronectin production by corneal fibroblasts and angiogenesis, as well as regulating the proliferation, migration, and apoptosis of certain fibroblasts and epithelial cells [14]. Furthermore, TGF-β1 promotes the differentiation of fibrocytes toward the myofibroblast phenotype, reinforcing the injured corneal tissue, but at the same time reducing corneal transparency due to the production of a disordered extracellular matrix and diminished crystallin protein production [15]. Interestingly TGF-β target protein expression occurs through different pathways in corneal keratocytes and corneal epithelial cells. For example, thrombospondin-1—a multifunctional matrix glycoprotein, which is highly expressed in the wounded cornea, is regulated through Smad signaling in epithelial cells, while in corneal fibroblasts it involves different TGF-β signaling pathway—probably by p38MAPK pathway. Moreover p15ink4b—a member of the inhibitors of cyclin-dependent kinase 4 family, which is also highly expressed in a debridement wound—is similarly regulated by TGF-β1 through the non-Smad signaling pathway in corneal epithelial cells. Whereas cellular fibronectin, which is also upregulated in the wounded cornea, is regulated through the Smad pathway in corneal fibroblasts [16].

### 2.2. Vascular Endothelial Growth Factor

The Vascular Endothelial Growth Factor (VEGF) family consists of a number of proteins involved in angiogenesis, including the most potent and widespread proteins VEGF-A, VEGF-B, VEGF-C, VEGF-D, VEGF-E, and Placental Growth Factor [17]. VEGF-A is a 32–42 kDa glycoprotein with at seven homodimeric isoforms with different solubility in the extracellular matrix. VEGF cell surface receptors (VEGFR-1 and -2) are expressed on many different cell types but mainly on endothelial cells. VEGF secretion is induced by cytokines and growth factors such as transforming growth factor alpha (TGF-α) and beta (TGF-β), epidermal growth factor (EGF), hepatocyte growth factor (HGF), basic fibroblast growth factor (bFGF), PDGF-BB, and interleukin-6, and it takes place in various phases of wound healing by numerous cells, of which the first to release VEGF are thrombin-activated platelets. VEGF takes part in wound healing processes by stimulating the migration of monocytes and their conversion into macrophages, vasodilatation, and increased vascular permeability through the induction of NO and prostacyclin release, migration, and the proliferation of endothelial cells, as well as endothelial stabilization and the formation of new vessels [18]. VEGF-B is a VEGF-A homolog that was observed to take part in corneal nerve fiber regeneration, sensation restoration and epithelial wound healing through reactivation of PI-3K/Akt-GSK-3β-mTOR signaling, neuronal oxidative stress regulation, and pigment epithelial-derived factor (PEDF) secretion [19]. Interestingly VEGF-B has been shown to have both pro- and anti-angiogenic properties. In cases of high FGF2/FGFR1 expression, VEGF-B inhibits its pathway, consequently preventing excessive angiogenesis [20].

### 2.3. Epidermal Growth Factor

Epidermal growth factor (EGF) is a peptide ligand of the transmembrane tyrosine kinase receptor—epidermal growth factor receptor (EGFR). The binding of EGF to EGFR results in homo- or heterodimerization, phosphorylation, and the recruitment of intracellular proteins. Then phosphorylated tyrosine residues bind to cytoplasmic signaling proteins, such as phospholipase C or phosphatidylinositol 3-kinase, inducing the migration, proliferation, and differentiation of cells [21]. In corneal epithelium EGFR is located primarily along the plasma membrane in the basal epithelial layers, with a decreasing concentration as the cells differentiate. In a wounded tissue, EGFR undergoes internalization and moves from the plasma membrane to cytosol through ligand binding. In normal conditions EGF is present in human tears at a concentration of approximately 2 ng/mL, a quantity sufficient to occupy approximately half of the EGFRs [22]. Following injury EGF promotes proliferation of epithelial cells equally in the limbus and peripheral cornea. It acts synergistically with IL-1 resulting in the chemotactic migration of cells and wound healing and is antagonized by TGF-β, which inhibits epithelial cell proliferation [23].

### 2.4. Platelet-Derived Growth Factor

Platelet-derived growth factor (PDGF) is another GF involved in the cornea regeneration process. PDGF signaling system is composed of four functional GFs—PDGF-AA, PDGF-BB, PDGF-CC, and PDGF-DD and their corresponding tyrosine kinase receptors—PDGFRα and PDGFRβ. Each of the aforementioned GFs is made up by polypeptide chains PDGF-A, -B, -C, and -D. Activation of the receptors occurs through dimerization or oligomerization of receptors monomers. Activated receptors interact with SH2-domain-containing signaling proteins—such as phospholipase C; the kinases Src, Fer, and PI3-kinase; the phosphatase SHP2; and adaptor proteins—resulting in the activation of signaling pathways including MAP kinase pathways, PI3-kinase-Akt, and PLCγ [24]. In corneal regeneration PDGF-BB seems to play the most significant role. PDGF-BB is produced by corneal epithelial cells and has been shown to promote nonfibrotic wound healing through the increase in cellular motility, autocrine secretion of TGFβ1, and the promotion of cell proliferation, as well as nonfibrotic matrix proteoglycans synthesis [25]. Moreover, synergistically with TGFβ, PDGF promotes myofibroblast differentiation in the corneal stroma following injury [26].

### 2.5. Nerve Growth Factor

Nerve growth factor (NGF) is one of the most common members of the neurotrophin family. NGF is mostly produced and released by nerve cells and is involved in the development and maintenance of the central and peripheral nervous systems. However, NGF also regulates immune cell functioning, inflammation, and wound healing process [27]. NGF and its receptors are widely expressed in the human cornea. It has been observed that corneal epithelial injury induces a significant increase in corneal NGF levels. Through binding to the NTRK1 receptor, it promotes cell survival and migration. Moreover it supports healing of corneal stroma and endothelial cells, as well as improving corneal sensitivity and tear function in patients with neurotropic keratitis [28].

### 2.6. Insulin-like Growth Factor (IGF)

The IGF family consists of insulin and the ligands IGF-1 and IGF-2, which are structurally similar to insulin, their cell surface receptors, six IGF binding proteins, and their proteases, as well as intracellular signaling proteins. IGF is active at the endocrine, paracrine, and autocrine levels and is crucial in the regulation of cell growth, proliferation, and survival [29]. In the human cornea IGF receptors are present in the epithelium, stromal keratocytes, and endothelium; in all of these corneal layers IGF has been shown to have significant functions in terms of wound repair. IGF-1 promotes corneal epithelial cell proliferation through the activation of Hybrid-R and the phosphorylation of Akt, and the migration and expression of Lamin-5 and β1-integrin. Moreover, it is suggested that IGF-1 induces differentiation of limbal stem cells into corneal epithelial cells and acts synergistically with Substance P in order to promote wound healing through corneal epithelial cell attachment to fibronectin and type IV collagen. In corneal stroma IGF-1 promotes keratocyte differentiation and the expression of the junction protein N-cadherin, while IGF-2 promotes keratocyte proliferation and collagen deposition without inducing myofibroblast differentiation [30].

### 2.7. Fibroblast Growth Factor

Fibroblast growth factor (FGF) is a diverse group of growth factors that influence a broad spectrum of cellular functions, such as cell migration, proliferation, differentiation, metabolism, and survival [31]. FGF plays an important role in tissue repair and regeneration. It induces dedifferentiation of cells, suppresses apoptosis, induces angiogenesis, and regulates protease expression and inflammation [32]. In the cornea, following injury, FGF induces the transformation of keratocyte into active fibroblasts, enhances the cell proliferation of limbal epithelium and stem cells, and inhibits hypoxia [33].

### 2.8. Substance P

Substance P is a neuropeptide that is secreted from sensory nerve endings and immune cells during inflammation. It plays a crucial role in neurogenic inflammation as it induces vasodilatation, microvascular permeability, plasma extravasation, and tissue edema, as well as stimulating macrophages to release inflammatory mediators [34]. However, it is also essential for maintaining corneal homeostasis and the corneal wound healing process by influencing cytokine and chemokine production. It acts synergistically with IGF-1 through the NK-1 receptor system, stimulating epithelial migration and increasing the attachment of corneal epithelial cells to fibronectin, collagen type IV, and laminine, maintaining the integrity of corneal epithelium. Moreover, substance P, activated through tyrosine kinase and protein kinase C pathways, induces corneal remodeling and healing through multiple molecules such as integrins, zonula occludens-1, focal adhesion kinase, paxillin systems, E-cadherins, almodulin-dependent protein kinase, and p38 mitogen-activated protein kinase [35]. It has been shown that substance P activates Akt and scavenges reactive oxygen species (ROS) through the NK-1 receptor, which inhibits the hyperosmotic stress-induced apoptosis of corneal epithelial cells [36]. In addition to affecting corneal epithelial cells, substance P also enhances keratocyte migration, inducing actin cytoskeleton reorganization and focal adhesion formation via the activation of phosphatidylinositide 3-kinase and the Ras-related C3 botulinum toxin substrate 1/Ras homolog gene family, member A pathways, as well as the upregulated expression of interleukin-8 [37].

### 2.9. Vitamin A

Vitamin A is crucial for maintaining the ocular surface homeostasis. It prevents the apoptosis of corneal epithelial cells by upregulating anti-apoptotic gene BCL-2 and reversing the pro-apoptotic gene BAX. Moreover, it promotes corneal wound repair by inducing cell adhesion and migration by regulating lysyl oxidase-like 4 [38]. Moreover it has been shown to decrease lysosomal membrane stability, increase macrophage influx and activation, and stimulation of collagen synthesis and reepithelization [39]. In rats vitamin A eye drops significantly improved the healing of corneal alkali burns and activated thrombospondin 2, reduced the sprouting of new vessels, and the production of VEGF-A, MMP 9, and TGF-β [40].

### 2.10. Fibronectin

Adhesion of the plasma protein called fibronectin is a significant component of the extracellular matrix. Injury induces its expression, which fills the bed of the lesion leading to the increased migration of corneal fibroblasts. It regulates integrins and is necessary for the contraction of stromal wounds during corneal repair [41,42].

**Figure 1:**
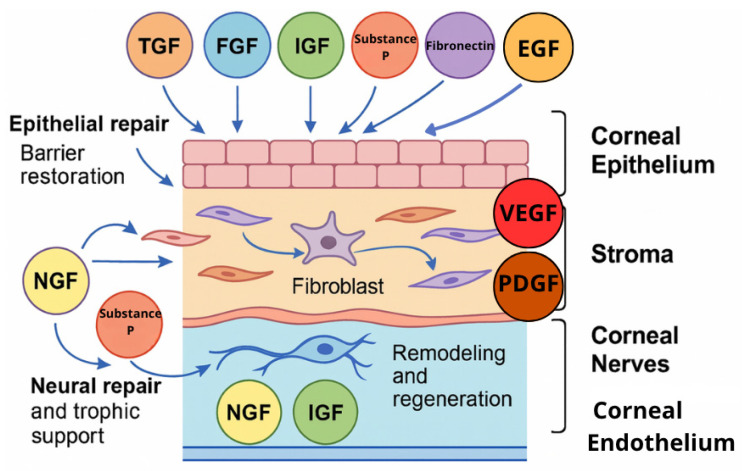
Interactive network of growth factors in ocular surface repair.

**Figure 2:**
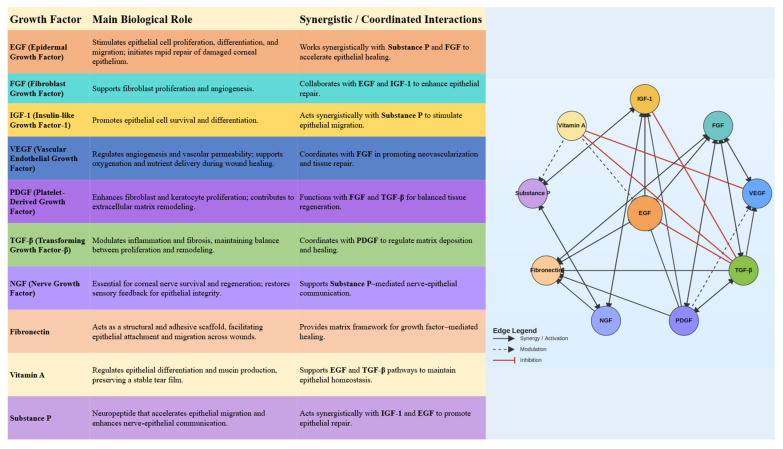
Interactions between growth factors in blood derivatives.

The ocular surface represents a complex and delicately balanced environment where epithelial cells, nerves, and extracellular matrix components interact to maintain transparency and integrity. Blood-derived eye drops provide a biologically active milieu that closely replicates the natural tear film. They contain multiple growth factors—including EGF, FGF, IGF-1, VEGF, PDGF, TGF-β, NGF, fibronectin, vitamin A, substance P, and cytokines—that function as an interactive signaling network (Figure 1). Their coordinated and synergistic actions stimulate epithelial proliferation and migration, modulate inflammation, and promote extracellular matrix remodeling (Figure 2). This complex interplay among growth factors ensures a balanced regenerative response, facilitating restoration of the corneal and conjunctival epithelium in ocular surface disorders such as severe dry eye, persistent epithelial defects, and post-surgical epithelial damage.

## 3. First-Generation Blood Derivatives: Serum-Based Therapies

The earliest group of blood-derived therapies comprises serum-based eye drops, originally produced from the patient’s own blood, and later also from a donor’s blood and umbilical cord blood. Developed in the late twentieth century, these formulations, based on a cell-free, fibrinogen-depleted fraction of blood obtained after clotting, gained attention because their biochemical profile resembles that of natural tears, providing molecules essential for ocular surface tissue integrity and homeostasis.

**Figure 3:**
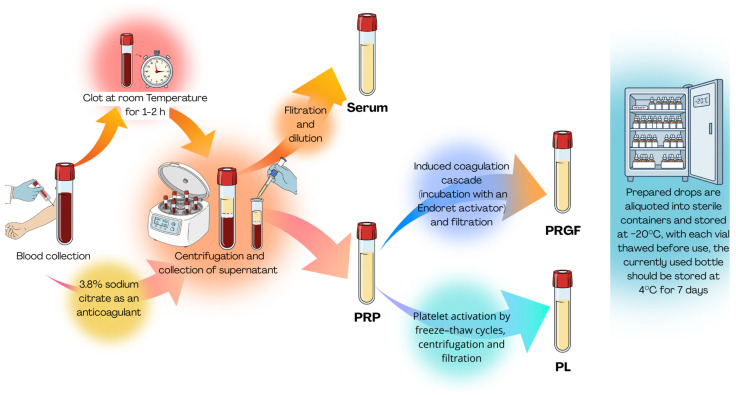
Blood derivative production.

The preparation process of the discussed blood-derivatives is presented in Figure 3.

### 3.1. Autologous Serum

The use of blood derivatives in ophthalmology was first described by Ralph et al. in 1975, who studied the effect of continuous delivery of fluids, including autologous serum (AS) or plasma, with a mobile perfusion pump in patients with damaged corneas [43]. Then, in 1984, Fox et al. reported the improvement of subjective and objective symptoms in patients with keratoconjunctivitis sicca after autologous serum eye drop treatment [44]. Subsequently, the popularization of the blood-derived topical therapy for the eye surface was contributed to by Tsubota et al., who demonstrated the stability of AS active components, as well as its efficacy and safety in the treatment of dry eye in Sjögren’s syndrome [45] and persistent corneal epithelial defect [46].

AS is similar to natural human tears not only in terms of pH (7.4) and osmolality (tears 298, serum 296 mOsm/L), but also in terms of growth factors including EGF and TGF-β, vitamin A, lysozyme, immunoglobins, and fibronectin, some of which appear in higher concentrations than in natural tears. It is to them that the serum owes its promoting effects on growth and migration and, at the same time, therapeutic effect on ocular surface disorders [47].

AS eye drops are prepared from patients’ own peripheral blood under sterile conditions. Qualification for therapy with autologous blood-derived preparation requires the exclusion of contraindications related both to blood collection and to the subsequent use of the blood-derived therapy. These contraindications include, among others, not signing an informed consent form, pregnancy or breastfeeding, acute or chronic systemic infection, any confirmed infectious disease or positive viral markers (HBV, HCV, HIV, or syphilis), cancer, hematologic disorders or blood-borne diseases, chronic liver or kidney disease, anticoagulant or antiplatelet therapy, significant cerebrovascular or cardiovascular disease, and anemia [48,49]. Protocols for the preparation of AS eye drops vary in terms of the tests carried out on patients, the time of blood clotting, the force and time used for centrifugation, depending on the medical center where they are prepared. These differences may affect the biochemical properties of the obtained eye drops [50]. For example, using a long clotting time (≥120 min) has been shown to result in higher concentrations of EGF, TGF-β1, and HGF, as well as improving effects on cell migration and differentiation, while increasing the g force of centrifugation from 500× *g* to 3000× *g* leads to a reduction in TGF-β1levels, while raising the levels of EGF and vitamin A, and inducing greater cell proliferation [51].

Another important factor in the preparation of AS eye drops is their concentration and the used diluents. In previously conducted studies, concentrations from 20% to 100% were used, with 20% dilution being the most common in controlled randomized trials conducted so far [52,53,54,55,56]. The possible toxicity of the serum on ocular surface cells supports the decision to dilute it to 20%. The concentration of TGF-β in human serum is five times higher than in human tears. This molecule has antiproliferative properties, and its high concentration may suppress wound healing of the ocular surface epithelium [57]. However, concentrations of 50% and 100% have also proved to be effective. In the study conducted by Cho et al., it was shown that in comparison with 50% AS diluted with saline and preservative-free artificial tear drops containing sodium hyaluronate and antibiotics, 100% AS with no dilution had significantly better effects on decreasing dry eye symptoms and corneal epitheliopathy in Sjögren’s syndrome as well as increasing the healing speed in persistent epithelial defects [58]. In another study, the effectiveness of 20% and 50% AS were compared in the treatment of dry eye. It was found that both concentrations worked equally and significantly in moderate dry eye, but in severe dry eye, a concentration of 50% was significantly better at alleviating dry eye symptoms [59].

The permissible shelf life of AS eye drops after preparation varies depending on the medical center, ranging from 3 to 12 months frozen and 24 h to 1 week thawed [60]. In the study conducted by López-García et al., it was shown that storage for 9 months at −20 °C does not affect the concentration of growth factors present in AS [61]. Concentrations of TGF-β1, EGF, and IGF-1 in 20% human serum were also shown to be stable up to one week of storage at 4–8 °C [62]. Another method of storage, like lyophilization, was also shown to preserve the properties of the AS, as no difference was found in the concentration of growth factors between fresh and lyophilized AS after 30 days of storage at 4 °C [63].

In vitro studies conducted on human eye surface cells showed that serum supports cellular mechanisms of tissue regeneration. Hardwig et al. showed that the incubation of SV-40 immortalized human corneal epithelial cells in serum resulted in a significantly higher proliferation rate as well as improving the migration and differentiation of cells in comparison with fresh frozen plasma [64].

Due to its properties in the healing and regeneration of ocular surface tissues, AS has been used in the treatment of ocular surface diseases as an alternative form of therapy to standard treatments.

The efficacy of AS eye drops in dry eye disease (DED), in comparison with conventional therapies such as artificial eye drops, was studied in several randomized controlled trials (RCT) with promising results (Table 1). In the first RCT conducted by Tananuvat et al. in 2001 [52], twelve patients with DED showed significant improvement in symptoms, objective signs, fluorescein and rose bengal staining, and conjunctival impression cytology in eyes treated with 20% AS. However, there was no statistical difference between the treatment and control groups [52]. A crossover trial by Noble et al. included 11 patients with DED who were randomized to receive 3 months of 50% autologous serum followed by 3 months of conventional treatment, or 3 months of their conventional treatment, followed by 3 months of autologous serum. Significant improvement in subjective symptoms was noted in patients treated with AS. Moreover, impression cytology analysis showed a statistically significant difference between eyes treated with conventional therapy and AS in favor of AS [48]. Kojima et al. compared 20% AS to artificial tears in 37 eyes of 20 patients with severe DED. Although there were no differences in terms of Schirmer test results between the two groups, in the group treated with AS tear film breakup time (TBUT), mean ocular surface staining scores, and pain scores showed significant improvement [53]. Similarly, a study by Noda-Tsuruya et al., which assessed 54 eyes of 27 patients with DED after LASIK, showed prolongation of TBUT and reduction in rose bengal staining score but no improvement in the Schirmer test value [65]. Another study conducted by Urzua et al. reported not only an improvement in TBUT and fluorescin staining score, but also a significantly greater relief in subjective symptoms reflected as OSDI score after short-term use of 20% AS compared to artificial tears as a control group in non-Sjögren DED patients [54]. A significantly greater decrease in OSDI score and prolongation of TBUT after a 1-month treatment with AS was also noted in a study conducted by Celebi et al.; however, differences between AS and artificial tears in terms of Schirmer’s test results and median OXFORD cornea and conjunctiva staining scores were not statistically significant [55]. Semeraro et al. reported that treatment with AS for 1 year in comparison with artificial tears resulted in significantly fewer sub-basal nerve branches and bead-like formations in in vivo confocal microscopy, as well as lower OSDI scores, but no improvement in clinical data (tear production, tear stability, ocular surface staining, conjunctival inflammation, and corneal thickness) was found [66]. A significant improvement in terms of TBUT and OSDI score was also noted after 1 month and 2 months of AS therapy in patients with DED after systemic isotretinoin therapy [67]. The most recent randomized controlled trial conducted by Zheng et al. included 232 participants—116 treated with AS and 116 with artificial tears for 12 weeks. Although improvements were noted in OSDI score, TBUT, Schirmer I test, corneal fluorescein staining, and conjunctival impression cytology for both groups, treatment with AS eye drops showed significantly better results in all of the tests [56].

To assess the quality of evidence supporting AS eye drops in DED, several systematic reviews and meta-analyses were performed. A 2017 systemic Cochrane database review analyzed five RCTs published by 5 July 2016, which compared 20% AS eye drops to artificial tears or saline in patients with dry eye of various origins. A total of 149 eyes of 92 participants were included. The study suggested that 20% AS can alleviate patient-reported symptoms of dry eye for up to 2 weeks, but there was no evidence of improvement over longer periods of time. However, meta-analysis could not be performed by the authors due to heterogeneity in the time points at which primary and secondary outcomes were reported and insufficient reporting of descriptive statistics [11]. A meta-analysis conducted by Franchini et al. included 10 RCTs with 353 patients. The study found that AS eye drops improve TBUT and OSDI scores in the short-term, but there was no significant difference between AS and artificial tears in terms of the Shirmer test and fluorescein staining scores. The evidence supporting this data was of low or very low quality due to inconsistency, imprecision, and risk of bias in most of the studies selected by the authors [68]. Analysis of seven RCTs by Wang et al. suggested that treatment with AS eye drops may also provide improvement in terms of rose bengal staining score, but the quality of the evidence was very low [69], which was also found by Quan et al. in their systematic review on this subject [70]. The results of the above-mentioned studies are largely congruent with each other. Findings from the network meta-analysis by Zhang et al. indicated that AS significantly improves tear film stability, as reflected in improvement in TBUT compared with AT. These effects may be mediated by the anti-inflammatory cytokines present in the serum, such as TGF-β and IL-1RA. However, AS did not significantly enhance tear secretion in the Schirmer test. The authors also noted that differences in preparation protocols, including dilution ratios, may substantially influence therapeutic efficacy [71]. The most recent meta-analysis conducted by Mederle et al. similarly showed that AS was more effective than AT in improving several DED parameters, including TBUT and Schirmer test scores. Nonetheless, improvements frequently failed to exceed the minimal clinically important difference, raising questions about their clinical relevance. Considerable heterogeneity across studies with regard to patient characteristics, disease severity, and preparation methods, further complicates interpretation and limits comparability [72].

Although DED remains the condition for which autologous serum eye drops have been used most frequently, their effects were also studied in other ocular surface diseases. Jeng et al. reported that 50% AS eye drops, administered every 2 h while awake, were successful in healing persistent corneal epithelial defects in eyes that were recalcitrant to conventional therapy [73]. In a study conducted by Lekhanont et al., 100% AS eye drop treatment in patients with corneal epithelial defects after various types of ocular surgery resulted in complete corneal epithelization in 170 of 181 patients over a median time of 4 days [74]. Non-diluted autologous serum was also studied in patients with persistent epithelial defects of the cornea by Lekhanont et al. In their study, complete corneal epithelization was observed in 95 of 109 eyes (87.16%) in patients who received 100% AS, and in 55 of 79 eyes (69.62%) in the control group. The median time to complete corneal epithelization was 14 days in the AS-treated group, while it was 28 days in the control group [75]. Similarly, an RCT by Kamble et al. showed that AS treatment facilitates faster reepithelialization of corneal epithelial defects after keratoplasty in comparison to artificial tears [76]. The efficacy of AS in corneal persistent epithelial defects was also reported in studies conducted by Lee et al. and Chen et al.; this time the 20% dilution of AS combined with silicone contact lenses resulted in the recovery of corneas after, respectively, 3 and 2 weeks [77,78]. The positive effects of AS on corneal epithelial healing were observed after various eye procedures such as pterygium surgery [79], photorefractive keratectomy [80], and epithelium-off corneal collagen crosslinking [81]. Additionally, AS has been shown to reverse severe contact lens-induced limbal stem cell deficiency [82]. Furthermore, AS eye drops have been successfully used in the treatment of neurotrophic keratopathy. A meta-analysis conducted by Roumeau et al., encompassing six studies involving 131 patients with neurotropic keratopathy treated with AS eye drops, found that AS effectively supports corneal healing, with results comparable to nerve growth factor eye drops, neurotization, and amniotic membrane transplantation [83].

Autologous serum eye drops are characterized by a good safety profile overall, with high toleration rates and rare adverse events. Although not frequent, complications associated with AS eye drop usage may occur; the most common of these include discomfort, ocular irritation, redness, itching, and discharge of mild intensity [9,52,56]. Other side effects have also been described in the literature. Welder et al. presented a case report of a patient with atopic keratoconjunctivitis who developed bilateral limbitis 4 weeks into 20% AS eye drop treatment, which resolved shortly after the discontinuation of the therapy [84]. There was also a case of a patient with a history of corneal transplant who developed a ringlike immunoglobulin deposition after treatment with AS eye drops [85]. Complications may also result from the contamination of the eye drops, as was seen in a patient with hypovitaminosis A who had severe bilateral Haemophilus influenzae keratitis following corneal perforation in one eye. The pathogen was isolated in the AS bottle used by this patient [86].

**Table 1:** Randomized controlled trials (RCTs) on topical use of autologous serum in treating ocular surface disorders.

Reference	Year	Study Design	Study Group (*n*)	Control Group (*n*)	Treated Condition	Intervention	Comparator/Control	Preparation	Dosage (Times/Day)	Duration	Outcomes Measured	Outcomes Significantly Improved Compared to Control
Tananuvat et al. [52]	2001	Paired-eye RCT	12	12	severe DED	AS	Saline solution	40 mL whole blood → centrifugation at 4200 rpm for 15 min → serum extraction → dilution to 20% with unpreserved normal saline solution	6	2 months	Subjective symptoms, OSS, TBUT and ST	Not significant
Noble et al. [48]	2004	Crossover RCT	16	16	DED (*n* = 11), other ESD (*n* = 5)	AS	AT	Whole blood → clot at 4 °C for 2–3 days → centrifugation → dilution to 50% with sterile normal saline	Vary	3 months	VAS, OSS, and IC	VAS, IC
Kojima et al. [53]	2005	RCT	10	10	DED	AS	AT	40 mL whole blood → centrifugation at 1500 rpm for 5 min → dilution to 20% with saline	6	2 weeks	VAS, OSS, TBUT, and ST	VAS, OSS, TBUT
Noda-Tsuruya et al. [65]	2006	RCT	12	15	DED post-LASIK	AS	AT	Not given	5	12 weeks	Subjective symptoms, OSS, TBUT, and ST II	TBUT, OSS,
Schulze et al. [87]	2006	RCT	13	10	ED in diabetic patients post-pars-plana vitrectomy	AS	Hyaluronic acid	Whole blood → clot for 1 h → centrifugation at 4000 rpm for 15 min → dilution to 50% with base salt solution	hourly	until complete epithelial healing	Rate of healing of the epithelial defects	Rate of healing of the epithelial defects
Urzua et al. [54]	2012	Crossover RCT	12	12	severe DED	AS	AT	20 mL whole blood → clot for 2 h at room temperature → centrifugation at 3500 rpm for 5 min at 4 °C → collection of supernatant → dilution at 20% with 0.9% saline solution	4	2 weeks	TBUT, OSDI, OSS	OSDI
Celebi et al. [55]	2014	Crossover RCT	20	20	severe DED	AS	AT	10 mL whole blood → clot for 2 h at room temperature → centrifugation at 4000 rpm for 10 min at 4 °C → collection of 5 mL of supernatant → dilution at 20% with 0.9% saline solution	4	1 month	ST, TBUT, OSDI, OSS	TBUT, OSDI
Mukhopadhyay et al. [88]	2015	RCT	48 (UCS), 52 (AS)	44	severe DED in Hansen’s disease	UCS, AS	AT	UCS: Umbilical cord blood → clot → centrifugation at 1500 rpm for 5 min → dilution to 20% with sterile saline solution AS: whole blood → centrifugation at 1500 rpm for 15 min → dilution to 20% with sterile normal saline solution	6	6 weeks	McMonnies score, OSS, TBUT, ST, IC	CBS: ST, McMonnies score, TBUT, OSS and IC; AS: McMonnies score, TBUT, OSS and IC
Semeraro et al. [66]	2016	RCT	12	12	SS-related DED	AS	AT	AS: whole blood → clot for 24–48 h at 4 °C → centrifugation at 4000 rpm for 10 min → dilution to 50% with saline	5	12 months	TBUT, OSDI, ST, OSS, inflammation, central corneal thickness, confocal microscopy	OSDI, number of branches, and number of beadings
Yılmaz et al. [67]	2017	Crossover RCT	24	24	DED due to systemic isotretinoin treatment	AS	AT	20 mL whole blood → clot for 2 h at room temperature → centrifugation at 4000 rpm for 10 min at 4 °C → dilution to 40% with isotonic saline solution	Not reported	1 month	OSDI, TBUT, and ST	TBUT, OSDI
Kamble et al. [76]	2017	RCT	35 (UCS), 35 (AS)	35	ED post-keratoplasty	UCS, AS	AT	UCS: Umbilical cord blood → clot → centrifugation at 1800× *g* for 10 min → dilution to 20% with sterile balanced salt solution AS: whole blood → clot → centrifugation at 1800× *g* for 10 min → dilution to 20% with sterile balanced salt solution	6	until complete epithelial healing	Decrease in size of ED and rate of epithelial healing	Rate of healing of the epithelial defects, Decrease in size of the ED (UCS > AS)
Akcam et al. [80]	2018	RCT	30	30	ED post-photorefractive keratectomy	AS	AT	20 mL whole blood → centrifugation at 1500 rpm for 10 min → dilution to 20% with artificial tears	6	until complete epithelial healing	Rate of healing of the epithelial defects, BCVA	Rate of healing of the epithelial defects
Sul et al. [79]	2018	RCT	25	25	ED post-pterygium surgery	AS	AT	20 mL whole blood → clot for 1 h at room temperature → centrifugation at 3000× *g* for 10 min → dilution to 50% with artificial tears	8	until complete epithelial healing	Rate of healing of the epithelial defects, pain score, conjunctival inflammation, and recurrences	Rate of healing of the epithelial defects, pain score
Rodríguez Calvo-de-Mora et al. [89]	2021	Three-arm RCT	21 (AS), 21 (AlS), 21 (UCS)	N/A	severe DED	AS, AlS, UCS	N/A	UCS: Umbilical cord blood → centrifugation → dilution to 20% with solution of Sterile Irrigating Solution, BSS^®^ AS: whole blood → centrifugation → dilution to 20% with solution of Sterile Irrigating Solution, BSS^®^ AlS: whole blood form AB-blood donors → centrifugation → dilution to 20% with solution of Sterile Irrigating Solution, BSS^®^	5	3 months	ST, TBUT, OSS	No significant differences were found
Zheng et al. [56]	2023	RCT	116	116	DED	AS	AT	40 mL whole blood → centrifugation at 3000 rpm for 15 min → dilution to 20% with saline solution	4	12 weeks	OSDI, TBUT, ST, OSS, IC	OSDI, TBUT, ST, OSS, IC
Kumari et al. [59]	2023	RCT	22	22	moderate and severe DED	50% AS	20% AS	20 mL whole blood → clot for 45 min at room temperature → centrifugation at 4000 rpm for 10 min → dilution to 20% or 50% with sterile 0.9% saline solution	6	12 weeks	OSDI, TBUT, OSS, ST	in severe DED—ST, TBUT, and OSS
Bachtalia et al. [90]	2025	RCT	10	10	SS-related DED	AS	AT	whole blood → clot for 2 h at room temperature → centrifugation at (4500 rpm) 3000× *g* (4500 rpm) for 15 min → dilution to 50% with artificial tears	4	3 months	TBUT, ST, OSS, improvement in the structure of the central corneal sub-basal nerve plexus	TBUT, ST, OSS, improvement in the structure of the central corneal sub-basal nerve plexus

Although AS eye drops have positive effects on eye surface diseases, there are several limitations to their use. Due to various conditions, such as cardiovascular disorders, infections, or poor venous access, not every patient is eligible for a blood donation. Moreover, their autologous character results in varied composition depending on a patient whose blood is used to prepare the product. Systemic, inflammatory, or autoimmune diseases may result in a higher content of proinflammatory cytokines that suppress regenerative properties and induce irritation. Chmielewska et al. reported that coexisting inflammatory processes may elevate IL-1β, IL-6, IL-10, and VEGF, and lower the IL-2 values of the serum, which negatively affects the therapeutic process and leads to adverse reactions including sand under the eyelids, impaired visual acuity, and worse eye lubrication [91]. AS from patients with systemic autoimmune diseases has been shown to have significantly lower levels of EGF and was less effective at lowering OSDI scores compared to AS in patients with localized ocular surface disease [92]. Furthermore, Kang et al. found that, in patients with chronic renal failure on hemodialysis, serum levels of EGF, PDGF-AB, and TGF-β1 were significantly lower than in healthy donors. Tests on a cell culture model showed that serum from these patients was less stimulating of the proliferation and migration of human corneal epithelial cells compared to serum from healthy donors [93]. Similarly, Harloff et al. showed that eye drops made from healthy donors’ serum contained significantly higher amounts of fibronectin and TGF-beta1, and induced higher stimulation of the proliferation and migration of human corneal epithelial cells than serum from immunosuppressed patients with rheumatoid arthritis [94]. These findings suggest that, in some cases, serum acquired from healthy donors may benefit patients with eye surface diseases more than AS.

### 3.2. Allogeneic Serum

Several limitations led to the introduction of allogeneic serum therapy. First of all, many patients with ocular surface disease suffer from systemic conditions like autoimmune disorders, anemia, or other blood disease which pose challenges in obtaining adequate blood volumes at regular intervals and can potentially alter the biological efficacy of the resulting preparation.

Autoimmune diseases such as Sjögren’s syndrome, rheumatoid arthritis, or systemic lupus erythematosus are associated with chronic inflammation and circulating autoantibodies that can alter the biochemical profile of the serum. Elevated levels of proinflammatory cytokines (e.g., TNF-α, IL-1β, and IFN-γ) and the presence of anti-epithelial or anti-nuclear antibodies may interfere with corneal epithelial healing and, in some cases, exacerbate inflammation on the ocular surface. Consequently, the therapeutic potential of autologous serum may be compromised or even counterproductive in such patients. In addition, in patients with severe systemic mucocutaneous disorders, such as Stevens–Johnson syndrome (SJS) or toxic epidermal necrolysis (TEN), the use of autologous serum is further contraindicated or unsafe.

Allogeneic serum obtained from healthy donors or umbilical cord blood seems to be a safe and effective alternative. It provides a standardized and readily available source of growth factors. Moreover, donor serums are screened for infectious diseases, prepared in large batches, and stored for extended periods under controlled conditions, ensuring consistent quality and availability.

Therefore, the shift toward allogeneic serum therapy in ocular surface disease management aims to overcome the limitations of autologous serum, improve treatment accessibility, and ensure a reliable, biologically active product for patients who cannot produce adequate autologous serum themselves.

Allogeneic serum is acquired from healthy donors’ peripheral blood and has been shown to be an effective alternative to AS. The guidelines for preparation protocols for allogeneic serum eye drops are in line with those for AS eye drops. Due to possible reactions between AB0 substances, HLA antibodies, and corneal epithelium, donors should be selected based on specific characteristics such as ABO-specific serum, male gender, and no history of blood transfusion [95]. Moreover, blood donors should be screened with standard tests performed by a blood bank and all blood donations should be tested for the presence of human immunodeficiency virus (HIV), hepatitis-B, hepatitis-C, hepatitis-E virus, and Treponema pallidum [96]. Some studies suggest that it may be advisable to perform additional tests for herpes simplex virus 1 and 2, cytomegalovirus, and varicella zoster virus. After collection, the serum should be quarantined for 4 months [97].

Allogeneic serum’s abilities to enhance tissue regeneration has been shown in a human epithelial cell culture model [98], as well as in clinical studies on patients with eye surface diseases such as persistent corneal epithelial defects [99] and dry eye [100] (Table 2). Retrospective collection of patient-reported outcome measures conducted by Lomas et al., which included responses from 279 patients with ocular surface diseases treated with either autologous (71) or allogeneic (208) serum, showed that both treatments led to a significant reduction in the severity of symptoms experienced by patients expressed as OSDI scores [101]. Moreover, a prospective, double-blind crossover trial conducted on 15 severe dry eye patients by van der Meer et al. showed no significant difference in terms of clinical outcomes including OSDI, TBUT, tear production, punctate lesions, and visual acuity between autologous and allogeneic serum [96]. Similarly, a randomized, double-blind clinical trial by Rodríguez Calvo-de-Mora et al. found that both autologous and allogeneic serum were effective in management of severe dry eye with comparable results in visual acuity scores, questionnaire scores, fluorescein staining scores, lissamine green dye, Schirmer test, and TBUT. In comparison to AS, allogeneic serum contained a significantly higher concentration of EGF but less IgA, IgG, and fibronectin. Interestingly, there were no statistical differences in efficiency of treatment between autologous and allogeneic serum eye drops in patients with autoimmune diseases [89]. Adverse events other than those reported for autologous serum have not been described in the literature, which is likely due to the rigorous screening procedures performed in donors prior to blood collection.

### 3.3. Umbilical Cord Serum

Another alternative to AS therapy for ocular surface diseases is the use of eye drops made of umbilical cord serum (UCS). This blood derivative is rich in growth factors, neurotrophic factors, and cytokines involved in the healing process, making it valuable in various fields of regenerative medicine, including ophthalmology [102]. Notably, the concentrations of key growth factors such as EGF, TGF-β, and NGF were found to be significantly higher in UCS compared to peripheral blood serum. This higher content of healing-promoting factors suggests that UCS eye drops could be more effective in treating ocular surface diseases [103,104].

Umbilical cord blood is acquired from healthy mothers immediately after uncomplicated cesarean section delivery after screening for parentally transmitted viral diseases, hepatitis B and C, human immunodeficiency virus, cytomegalovirus, and syphilis [105]. Then UCS is prepared under sterile conditions. Collected samples are allowed to clot for 2 to 4 h at room temperature and then centrifugated. Next, the serum is isolated into a new sterile tube, heat-inactivated at 56 °C for 30 min, and filtrated to remove donor-to-donor variations [102]. The first study to compare UCS eye drops with AS eye drops was conducted by Vajpayee et al. in 2003 [106] and included 60 eyes of 59 patients with persistent corneal epithelial defects. The study demonstrated a significantly greater decrease in the epithelial defect and a higher number of eyes achieving complete re-epithelization in the group treated with UCS compared to the AS group [106]. Since then, UCS eye drops have been shown to be effective in eye surface diseases such as persistent corneal epithelial defect [107] and dry eye of various origins [104], such as graft-versus-host disease [108], Stevens–Johnson syndrome [109], Hansen’s disease [88], and neurotrophic keratitis [103] (Table 3). In vivo confocal microscopy of the corneal subnasal nerve plexus in patients with dry eye revealed that both allogenic and cord blood serum improved nerve density, length, and width, and fractal dimension of corneal subnasal nerve plexus. However, UCS induced a more pronounced increase in the corneal nerve fractal dimension, indicating a greater degree of nerve regeneration [110].

A multicenter, randomized, double-masked, cross-over clinical trial conducted by Campos et al. compared the efficacy of UCS with allogeneic peripheral blood serum in 60 patients with severe DED that was resistant to conventional therapy. Both treatment options were shown to be effective, but UCS therapy had better results in terms of reducing corneal damage and decreasing symptoms. After 1 month of therapy, a significant improvement in corneal staining was observed in 53 out of 60 studied eyes (88.3%), with the UCS group demonstrating a notably greater difference between days 3–5 and day 30 of treatment. Alleviation of subjective symptoms expressed as OSDI scores was noted for both groups, as well as the Visual Analog Score and conjunctival staining. Interestingly, the authors found a direct relationship between the reduction in corneal damage and the levels of EGF, TGFα, PDGF, FGF, and IL13. They also observed an indirect relationship between IGF1 and IGF2, as well as a direct relationship between OSDI subset A and B reduction and IL13. Both treatments were evaluated as safe as no adverse events were recorded [111]. The superior healing potential of UCS over AS eye drops was also shown in an RCT by Kumar et al. across various patient groups. In patients with DED, the UCS-treated group showed greater improvement in visual acuity, eye sensation score, and OSDI by the 7th day, as well as better results in the Schirmer test, TBUT, and corneal fluorescein staining score by 3 months. Among patients with acute chemical burns, the UCS group exhibited more significant improvement in visual acuity, re-epithelization, corneal clarity, and reduction in limbal ischemia by 3 months. In patients with ocular allergy, the UCS group also showed an improvement in eye sensation score by the 7th day [112]. UCS was also found to be effective in acute ocular chemical burns. A double-blind prospective randomized controlled clinical trial conducted by Sharma et al. included 33 eyes of 32 patients with acute ocular chemical burns treated within 3 months after the injury, randomized into three groups receiving either 20% UCS, 20% AS, or artificial tear drops. UCS was shown to be more effective than AS and artificial tears in providing pain relief, promoting faster epithelial healing, reducing limbal ischemia, and improving corneal clarity in patients with acute ocular chemical burns [113]. Its efficacy was later presented in another RCT as comparable to amniotic membrane transplantation, but with the advantage of faster improvement in corneal clarity, better pain control, and the avoidance of surgery in an inflamed eye [114]. However, in the previously mentioned paper by Rodríguez Calvo-de-Mora et al., although UCS was found to be an effective treatment option in severe dry eye, it was not superior to autologous and allogeneic peripheral serum. Moreover, UCS contained a lower concentration of molecules such as EGF and TGF compared to autologous and allogeneic serum, which may have been related to the larger amount of time between the extraction of the umbilical cord blood and the preparation of UCS drops compared to autologous and allogenic serum eye drops [89]. No serious adverse events were reported in the literature. According to the network meta-analysis by Zhang et al., UCS may be particularly beneficial for patients with aqueous-deficient DED. UCS demonstrated significant improvements in TBUT when compared with AT, an effect potentially linked to its distinct GF profile, including placental growth factor. Although UCS did not significantly improve Schirmer test scores, SUCRA analysis indicated a possible advantage in promoting tear secretion relative to other blood derivatives [71].

**Table 3:** Randomized controlled trials (RCTs) on topical use of umbilical cord serum in treating ocular surface disorders.

Reference	Year	Study Design	Study Group (*n*)	Control Group (*n*)	Treated Condition	Intervention	Comparator/Control	Preparation	Dosage (times/Day)	Duration	Outcomes Measured	Outcomes Significantly Improved Compared to Control
Vajpayee et al. [106]	2003	RCT	30	29	PED	UCS	20% AS	Umbilical cord blood → clot → centrifugation at 1500 rpm for 5 min → dilution to 20% with sterile saline solution	6	3 weeks	Rate of healing of the epithelial defects	Rate of healing of the epithelial defects
Sharma et al. [113]	2011	RCT	12	10 (AT), 11 (AS)	acute ocular chemical burns (AOCB)	UCS	AT, 20% AS	UCS: Umbilical cord blood → clot → centrifugation at 1800× *g* for 10 min → dilution to 20% with sterile balanced salt solution AS: whole blood → clot → centrifugation at 1800× *g* for 10 min → dilution to 20% with sterile balanced salt solution	10	3 months	Pain score, size, and area of epithelial defect, extent of limbal ischemia, corneal clarity, and symblepharon formation	Pain score, rate of healing of the epithelial defect, limbal ischemia, corenal clarity, vascularization
Mukhopadhyay et al. [88]	2015	RCT	48 (UCS), 52 (AS)	44	severe DED in Hansen’s disease	UCS, AS	AT	UCS: Umbilical cord blood → clot → centrifugation at 1500 rpm for 5 min → dilution to 20% with sterile saline solution AS: whole blood → centrifugation at 1500 rpm for 15 min → dilution to 20% with sterile normal saline solution	6	6 weeks	McMonnies score, OSS, TBUT, ST, IC	CBS: ST, McMonnies score, TBUT, OSS and IC; AS: McMonnies score, TBUT, OSS and IC
Sharma et al. [114]	2016	RCT	15 (UCS), 15 (AMT)	15	AOCB	Standard medical therapy + UCS/AMT	Standard medical therapy alone	UCS: Umbilical cord blood → clot → centrifugation at 1800× *g* for 10 min → dilution to 20% with sterile balanced salt solution	10	3 months	Corneal clarity, rate of healing of the epithelial defects, pain score, BCVA, symblepharon, tear film status, and lid abnormalities	UCS: corneal clarity, pain score
Kamble et al. [76]	2017	RCT	35 (UCS), 35 (AS)	35	ED post-keratoplasty	UCS, AS	AT	UCS: Umbilical cord blood → clot → centrifugation at 1800× *g* for 10 min → dilution to 20% with sterile balanced salt solution AS: whole blood → clot → centrifugation at 1800× *g* for 10 min → dilution to 20% with sterile balanced salt solution	6	until complete epithelial healing	Decrease in size of ED and rate of epithelial healing	Rate of healing of the epithelial defects, Decrease in size of the ED (UCS > AS)
Campos et al. [111]	2019	Crossover RCT	31	29	severe DED, associated with PED	UCS	20% AS	UCS: Umbilical cord blood → centrifugation at 2800× *g* for 10 min → dilution to 20% with sterile phosphate-buffered saline AS: whole blood → centrifugation at 2800× *g* for 10 min → dilution to 20% with sterile phosphate-buffered saline	8	1 month	OSDI, OSS, TBUT, ST,	OSDI subset A score, OSS
Moradian et al. [105]	2020	RCT	40	40	ED in diabetic patients post-vitrectomy	UCS	AT	Umbilical cord blood → centrifugation at 1500 rpm for 5 min → dilution to 20% with preservative-free artificial tears	6	12 days	Rate of healing of the epithelial defects	Rate of healing of the epithelial defects
Rodríguez Calvo-de-Mora et al. [89]	2021	Three-arm RCT	21 (AS), 21 (AlS), 21 (UCS)	N/A	severe DED	AS, AlS, UCS	N/A	UCS: Umbilical cord blood → centrifugation → dilution to 20% with solution of Sterile Irrigating Solution, BSS^®^ AS: whole blood → centrifugation → dilution to 20% with solution of Sterile Irrigating Solution, BSS^®^ AlS: whole blood form AB blood donors → centrifugation → dilution to 20% with solution of Sterile Irrigating Solution, BSS^®^	5	3 months	ST, TBUT, OSS	No significant differences were found
Kumar et al. [112]	2023	RCT	20 (DED), 21 (AOCB), 20 (OA)	20 (DED), 21 (AOCB), 20 (OA)	DED, AOCB, OA	UCS	AS	Not given	Not reported	3 months	BCVA, eye sensation score (ESS), OSDI, TBUT, ST, OSS, epithelial defect, limbal ischemia, corneal clarity, and improvement in grade of severity	BCVA, eye sensation score, OSDI, ST, TBUT, and OSS (DED), BCVA, reepithelialization, reduction in limbal ischemia, and corneal clarity (ACB), improvement in ye sensation score (OA)

Taking all into account the higher level of proinflammatory cytokines IL-1β, IL-6, IL-10, VEGF, and TGF-B, together with uncontrolled presence of immonoglobulins and complement in autologous serum, may bring about harmful effects on the ocular surface. Given that the concentration of TGF-β in conventionally prepared 100% autologous serum is reported to be up to fivefold higher than in natural tears, and that dilution of the preparation to reduce TGF-β toxicity simultaneously decreases other beneficial trophic factors—thereby potentially diminishing the biological efficacy of the serum—a platelet-derived formulation may represent a more physiologically balanced and therapeutically advantageous alternative.

Platelet-rich plasma represents a more biologically active and functionally comprehensive alternative to autologous serum, with the additional advantages of easier preparation, a simpler processing protocol, and a shorter preparation time.

## 4. Second-Generation Blood Derivatives—Platelet-Rich Plasma

Platelet-rich plasma (PRP) is defined as a preparation of autologous plasma with a platelet concentration above the normal platelet count present in whole blood [115]. PRP is obtained from patients’ own peripheral blood. Whole blood is collected in vials with a anticoagulant to prevent platelet activation and then centrifuged to separate blood components. There are various protocols for PRP preparation, and methods such as the PRP method and buffy coat method, which differ in the number of steps, centrifugal acceleration, number of spins, time, and the distance between the particles and the rotor, resulting in variations in the concentration of platelets and leukocytes in the final product [116]. Beside cell adhesion molecules such as fibrin, fibronectin, and vitronectin, the high concentration of platelets in PRP is associated with being actively secreted by the growth factors involved in wound healing, including platelet-derived growth factor (PDGF), TGF-β1 and TGF-β2, VEGF, and EGF [117]. The PDGF contained in platelet alfa granules is crucial for tear stability and the maintenance of a healthy ocular surface; it has a chemotactic effect on fibroblasts, monocytes, and macrophages, and acts synergically with TGF-β to promote myofibroblast differentiation [118].

PRP was first introduced into hematology in the 1970s as a transfusion product for patients with thrombocytopenia. It was incorporated in maxillofacial surgery as a platelet-rich fibrin 10 years later, and has since then been used largely in musculoskeletal fields in sports injuries but also in other fields including ophthalmology [119]. One of the first studies to implement PRP in the field of clinical ophthalmology was conducted in 2007 by Alio et al., who reported that PRP eye drops are an effective form of treatment in dormant corneal ulcers, as they promote epithelial healing and improve inflammation and subjective symptoms [120].

Since then, several RTCs on PRP as a treatment for eye surface diseases have been conducted (Table 4). Panda et al. investigated the effectiveness of PRP eye drops as a supplementary treatment alongside standard medical therapy in acute ocular chemical injury. PRP was administered in 10 of 20 eyes included in the study, resulting in the statistically significantly faster healing of epithelial defects and better corneal clarity after 3 months of treatment. An improvement in best corrected visual acuity was also observed, but the difference was not statistically significant [121]. In the study conducted by Javaloy et al., PRP eye drops promoted epithelial status after LASIK but had no positive effect on corneal sensitivity [122]. Avila et al. found that PRP administered in the form of an injection into the lacrimal gland promoted a reduction in corneal staining, an increase in lacrimal production (presented as a mean Schirmer value and as TBUT), and an improvement in OSDI values in patients with severe dry eye [123]. García-Conca et al. compared the efficacy of PRP eye drops and artificial tears in 168 eyes of 84 patients with hyposecretory dry eye. The authors observed significantly greater reductions in OSDI scores, visual improvement, hyperaemia, and osmolarity, as well as an increase in calciform cell density, better Schirmer test and TBUT scores, and a more significant reduction in corneal and conjunctival staining in the group treated with PRP eye drops. The restoration of physiological levels of tear osmolarity and calciform density was more significant in eyes with greater ocular surfaces and visual degradation at baseline, which may indicate that PRP is more effective in eyes with moderate and severe dry eye [124]. The ability of PRP to improve healing of the corneal epithelium was also shown by Kamiya et al. in patients after phototherapeutic keratectomy. Although there were no differences between PRP and artificial tears groups on day 7 of the treatment, on days 1 and 2 the group treated with PRP showed a significantly smaller staining area and a higher recovery rate of the corneal epithelium, which indicates that PRP enhances epithelial recovery in the early postoperative period [125]. Several studies compared PRP and AS eye drops in terms of growth factor content and efficacy in the treatment of dry eye. Metheetrairut et al. measured that 20% PRP contained significantly higher concentrations of fibronectin and TGF-β1 as well as a trend towards a higher EGF concentration in comparison with 20% AS. Both blood derivatives improved scores of the two questionnaires used to measure subjective symptoms; post-treatment improvement was not significantly different in groups treated with PRP and AS. However, after 1 month of treatment, PRP significantly enhanced both measures, whereas AS showed a significant improvement only in OSDI scores. Although neither PRP nor AS significantly influenced TBUT, the Schirmer test, or the Oxford staining score, there was a significant difference between those two treatments in favor of PRP. Moreover, PRP significantly improved the best corrected visual acuity (BCVA) after 1 month of treatment. No adverse events were noted. The study also reports that PRP eye drops can be stored at −20 °C for 3 months without a decrease in the concentration of epitheliotropic factors [8]. Kang et al. reported that, in patients with dry eye related to primary Sjögren’s syndrome, 20% AS and 20% PRP were comparably efficient over a 12-week treatment period, as both improved corneal and conjunctival staining scores as well as TBUT, with no statistical differences between groups. Similarly to the previous study, there were no complications due to the treatment [126]. Wróbel-Dudzińska et al. evaluated that in comparison to AS, PRP contains significantly higher concentration of FGF, EGF, fibronectin, VEGF, PDGF AB, NGF, PDGF and substance P, while it contains a lower level of TGF-β which is known to suppress wound healing in high concentration. Authors compared efficacy of the treatment with either 100% AS or 100% PRP in patients diagnosed with dry eye disease in primary Sjögren’s syndrome. Both treatment options improved BCVA, Schirmer test, TBUT and ocular surface staining, and conjunctival hyperaemia. However, only the mean change in OSDI was statistically significant. Statistically significant differences between groups treated with PRP and AS were shown for OSDI and BCVA [49]. Conversely, another study reported that PRP showed lower concentrations of EGF and VEGF than AS, which may be due to a different preparation protocol [127].

The benefits of PRP therapy in dry eye disease were proved in several meta-analyses conducted by Kwaku Akowuah et al., as it presented a significant improvement in terms of subjective dry eye symptoms, tear quality and production, and corneal staining. The occurrence of adverse effects was 2.5% for traditional PRP and included itching, dizziness, conjunctivitis, eye pain, and irritation [128]. Zhang et al. reported that PRP showed the most pronounced effects on corneal fluorescein staining and OSDI scores, suggesting potent regenerative capacity and symptom relief. However, PRP did not significantly improve TBUT compared with AT, indicating that its therapeutic action may rely more on epithelial repair mechanisms than on stabilizing the tear film. As with other preparations, methodological inconsistencies were identified as key factors influencing treatment outcomes [71]. In the network meta-analysis by Mederle et al., PRP also demonstrated superior efficacy to AT. Importantly, the route of administration significantly affected results, as lacrimal gland injections led to greater improvements in Schirmer test results than topical PRP eye drops. Nevertheless, similarly to AS, many PRP-related improvements did not surpass the minimal clinically important difference. The authors additionally reported substantial heterogeneity across study designs, populations, and preparation techniques, which limits direct comparison between studies [72].

**Table 4:** Randomized controlled trials (RCTs) on topical use of platelet-rich plasma in treating ocular surface disorders.

Reference	Year	Study Design	Study Group (*n*)	Control Group (*n*)	Treated Condition	Intervention	Comparator/Control	Preparation	Dosage (times/Day)	Duration	Outcomes Measured	Outcomes Significantly Improved Compared to Control
Panda et al. [121]	2012	RCT	10	10	AOCB	PRP	AT	Not given	10	3 months	Corneal clarity, rate of healing of the epithelial defects, BCVA	Corneal clarity, rate of healing of the epithelial defects
Javaloy et al. [122]	2013	RCT	54	54	corneal sensitivity post-LASIK	PRP	Saline solution	Not given	6	3 months	OSS, corneal sensitivity, confocal microscopy of sub-basal nerve plexus	OSS
García-Conca et al. [124]	2019	RCT	44	39	DED	PRP	AT	40 mL whole blood → blood processing with RegenKit^®^ Ophthalmology PRPTM Preparation kit (Regen Lab USA LLC, New York, NY, USA) → centrifugation at 1500× *g* for 5 min → resuspension of the cell pellet in the supernatant	6	1 month	OSDI, OSS, TBUT, ST, and tear osmolarity	OSDI score, BCVA, hyperaemia, ST, TBUT, OSS, tear osmolarity
Kamiya et al. [125]	2021	RCT	5	5	ED post-phototherapeutic keratectomy	PRP	AT	30–32 mL whole blood with ACD-A as anticoagulant→ injection into a TriCell PRP Kit → centrifugation at 3200 rpm for 4 min → mixing plasma and buffy coat → centrifugation at 3300 rpm for 3 min → sewparation of PRP and platelet-poor plasma	6	2 weeks	Rate of healing of the epithelial defects, decrease in size of the ED, subjective symptoms	Rate of healing of the epithelial defects, Decrease in size of the ED
Metheetrairut et al. [8]	2022	RCT	10	10	DED	PRP	20% AS	AS: 35 mL whole blood → clot for 2 h at 31 °C → centrifugation at 3000× *g* for 30 min at 4 °C → dilution to 20% with balanced salt solution PRP: 35 mL whole blood with sodium citrate → centrifugation at 2200× *g* for 10 min at 22 ± 2 °C → dilution to 20% with balanced salt solution	10	1 month	ST, BCVA, OSDI, TBUT, OSS	ST and BCVA
Kang et al. [126]	2023	RCT	14	16	SS-related DED	PRP	20% AS	AS: 24 mL whole blood → clot for 2 h at room temperature → centrifugation at 3500 rpm for 15 min → dilution to 20% with 0.1% sodium hyaluronate preservative-free eye drops PRP: 22 mL whole blood with 3.2% sodium citrate → injection into a PRS Bio Kit → centrifugation at 3000× *g* for 3 min → mixing plasma and buffy coat → dilution to 20% with 0.1% sodium hyaluronate preservative-free eye drops	6	12 weeks	OSS, ST, TBUT, OSDI, IC metaplasia grade and goblet cell density grade	No significant differences were found
Jongkhajornpong et al. [10]	2024	RCT	48	48	DED	PRP	100% AS	AS: 50 mL whole blood → clot for 2 h at room temperature → centrifugation at 3000× *g* for 15 min PRP: 36 mL whole blood with 3.2% sodium citrate → centrifugation at 350× *g* for 10 min at 20 °C	8	4 weeks	OSDI, OSS, TBUT, ST, meibum quality and expressibility	No significant differences were found
Sachan et al. [129]	2025	RCT	20	20	DED	PRP	AT	10 mL whole blood with sodium citrate → centrifugation at 2400 rpm → separation of upper 2/3 → centrifugation at 3600 rpm → extraction of PRP and buffy coat → dilution to 20% with balanced salt solution	Not reported	3 months	OSDI, tear meniscus height, TBUT, ST, OSS, IC, BCVA	OSDI, tear meniscus height, TBUT, ST, OSS, IC

Injectable PRP is widely used in various fields of regenerative medicine due to its ability to deliver concentrated growth factors directly into target tissues. In ophthalmology, periocular or subconjunctival PRP injections have been employed for the treatment of severe dry eye disease, neurotrophic keratitis, persistent corneal epithelial defects, ocular surface burns, and post-surgical ocular surface repair (following photorefractive keratectomy (PRK), LASIK, or pterygium excision to enhance epithelial recovery).

Overall, PRP injections into the lacrimal gland and subconjunctival space represent a promising therapeutic strategy that combines regenerative potential with favorable safety and tolerability profiles.

When delivered as an intraglandular injection into the lacrimal gland, PRP has been shown to enhance residual acinar and ductal cell activity, increase aqueous tear secretion, and improve tear film stability. In a prospective study, lacrimal gland PRP injection in patients with severe dry eye secondary to Sjögren’s syndrome significantly improved Schirmer scores, tear breakup time, tear meniscus height, and corneal staining compared to untreated eyes [130].

Subconjunctival injection of PRP provides a localized delivery of these growth factors to the conjunctiva and cornea, creating a favorable microenvironment for wound healing and epithelial repair. TGF-β and IGF-1 are particularly important in reducing scarring and supporting extracellular matrix stabilization, while NGF promotes corneal nerve regeneration, improving corneal sensitivity and ocular surface homeostasis. This method has been applied successfully in cases of persistent epithelial defects, ocular surface disease following refractive surgery, chemical burns, and autoimmune keratoconjunctivitis sicca, where conventional therapies are often insufficient [131,132].

Subconjunctival PRP injection provides trophic support to the conjunctiva and adjacent ocular surface tissues, promoting epithelial regeneration, goblet cell recovery, and reduction in chronic inflammation. Long-term clinical benefit has been documented, with sustained improvements in the Ocular Surface Disease Index (OSDI), Schirmer’s test, and ocular surface staining up to 12 months after treatment [133].

According to the literature, reported side effects are typically mild, transient, and related to the injection procedure rather than the biological product itself. These include pain, erythema, or edema at the injection site (typically resolving within 24–72 h); subconjunctival hemorrhage or mild bruising; a transient foreign-body sensation or ocular irritation; and, rarely, infection if aseptic technique is not strictly maintained.

Serious complications such as corneal infection, intraocular inflammation, or fibrosis are exceedingly rare and are primarily linked to procedural errors rather than PRP composition.

Injectable PRP offers distinct biological and pharmacokinetic benefits such as deeper tissue delivery, higher local bioavailability and reduced dosing frequency, and sustained release of growth factors.

Overall, intraglandular and subconjunctival PRP injections represent a promising regenerative therapy for ocular surface disorders, offering both symptomatic relief and measurable improvements in ocular surface homeostasis, with only minor and transient adverse effects reported [134].

## 5. Third-Generation Blood Derivatives—Platelet Lysate and Plasma Rich in Growth Factors

Advances in processing techniques have led to the introduction of third-generation preparations, including platelet lysate (PL) and plasma rich in growth factors (PRGF). These products were designed to overcome some of the limitations of earlier blood derivatives by enhancing standardization, bioactivity, and safety. Owing to their concentrated trophic factor profile and the absence of intact platelets or leukocytes, these formulations have shown strong regenerative capabilities.

The controlled activation of platelets during PRGF preparation ensures a sustained and physiologically balanced release of growth factors, supporting gradual and prolonged biological activity compared to other platelet derivatives. Unlike platelet-rich plasma, platelet lysate exerts its therapeutic effects immediately upon application, as growth factors are already released and available in a soluble form. This eliminates the variability associated with platelet activation and release kinetics, resulting in a faster and more consistent biological response. Both products have anti-inflammatory and anti-fibrotic effects, primarily mediated by TGF-β and PDGF, contribute to controlled tissue remodeling, help modulate wound healing, and reduce scarring. Moreover, PRGF and PL, as leukocyte-free products, ensure excellent biocompatibility and safety, minimizing the risk of immune reactions, infection, and inflammatory complications.

### 5.1. Plasma Rich in Growth Factors

A type of PRP that has been researched frequently in recent years for its use in ocular surface diseases is PRGF. It is made from platelet-enriched plasma, where the platelets are degranulated to release their stored growth factors. After filtering, these eye drops are rich in biologically active components, including growth factors, neurotrophic agents, vitamin A, and fibronectin, while maintaining low levels of proinflammatory molecules [135]. In preclinical studies, PRGF was found to be as effective as AS in promoting the proliferation of human corneal epithelial cells. Additionally, it was shown to stimulate the proliferation of human keratocytes and human conjunctival fibroblasts, as well as the migration of human corneal epithelial cells, human conjunctival fibroblasts, and human keratocytes, to a greater extent than AS. Moreover, unlike AS, PRGF maintains human conjunctival fibroblasts and human keratocyte activity and reduces TGF-β1-induced myodifferentiation [136,137].

The efficacy of PRGF eye drops has been studied for various eye surface diseases. López-Plandolit et al., in a series of prospective noncomparative cases, reported that, in patients with persistent corneal epithelial defects, PRGF eye drop treatment resulted in the restoration of epithelial defects in 17 of 20 eyes (85%) with an epithelization time ranging from 2 to 39 weeks (a mean time of 10.9 weeks). The treatment was overall well tolerated with one exception, in which it was discontinued due to redness and itching of the eye [131]. The efficacy of PRGF eye drops was also evaluated in 16 patients with dry eye. PRGF improved dry eye symptoms, reducing modified SDEQ scores by <25% in 25%, by 25–50% in 31.25%, and by >50% in 43.75% of patients. Impression cytology observation showed that, in 62.5% of patients, PRGF treatment significantly reduced the signs of squamous metaplasia. Moreover, it led to a reduction in the need to use associated therapies [138]. The improvement of dry eye signs and symptoms, such as OSDI score, ocular redness, TBUT, Schirmer test, and BCVA, after PRGF treatment was also observed in evaporative dry eye [139], dry eye related to congenital aniridia [140], after LASIK surgery [141], as well as in patients with Sjögren syndrome [142] and graft-versus-host diseases [143]. Sanchez-Avila et al. suggested that PRGF eye drops may be a safe and effective treatment of patients with neurotrophic keratitis stages 2 and 3, as they resulted in the complete resolution of corneal defects in 37 of the 38 studied patients (97.4%) and statistically significant reductions in OSDI, VAS frequency, and VAS severity, as well as improvement in BCVA [144]. Furthermore, the alleviation of subjective symptoms after PRGF treatment was reported in patients with keratoneuralgia, since SANDE questionnaire scores improved by 40 points out of a 100-point scale, and 10 out of 16 patients (63%) reported that PRGF was by far the best treatment method they had used [145]. de la Sen-Corcuera et al. noted that PRGF reduces inflammation and disease severity in eyes with cicatrizing conjunctivitis. Interestingly, the authors observed that the treatment resulted in the significant improvement of intraocular pressure [146], which was also observed by Sánchez-Avila et al. in a retrospective case series study on secondary ocular surface disorders in patients with glaucoma [147]. The efficacy and safety of PRGF in ocular surface diseases was reported in a multicenter interventional case series conducted by Soifer et al. The study included 153 patients with eye surface diseases including dry eye, neurotropic keratopathy, dormant corneal ulcers, limbal stem cell deficiency, and cicatrizing conjunctivitis. The authors concluded that PRGF treatment significantly improved patient-reported symptoms measured by the SANDE score, regardless of the etiology. Furthermore, PRGF reduced objective signs. Not only did it significantly decrease the presence of punctate epithelial erosions and persistent epithelial defects but it also resolved corneal signs completely in 47.5% and partially in 31.2% out of 61 dry eye cases. Additionally, 67% of persistent epithelial defects were observed to resolve within 3 months. The treatment was evaluated as well tolerated, with only one patient reporting an adverse event in the form of a burning sensation [148].

It is also worth mentioning that PRGF eye drops are suitable for long-term use, as they can be stored for up to 12 months at −20 °C and 7 days at 4 °C or at room temperature without a decrease in the main growth factors and proteins or any microbial contamination [149].

### 5.2. Platelet Lysate

Platelet lysate is obtained by activating PRP in a freeze–thaw process. First, the platelet concentrate is frozen at −80 °C to −30 °C and then thawed at 37 °C to break up the platelets and release growth factors. This process may be performed up to five times, according to studies conducted so far [150]. In comparison to autologous and allogenic serum, PL has been showed to contain higher concentrations of TGF- β1, PDGF-AB, PDGF-BB, FBG, and EGF, but less IGF-1, HGF, and fibronectin [151]. Several in vitro studies have been conducted on PL’s influence on corneal cells. The ability of PL to enhance corneal epithelial cell proliferation and migration was shown to be comparable to that exerted by human peripheral serum [152]. Furthermore, PL has been shown to promote corneal endothelial cell proliferation and viability, as well as to increase endothelial cell markers [153], which was also reported for purified human platelet pellet lysate. Moreover, purified human platelet lysate also enhanced corneal endothelial cell migration and exerted antioxidative and antiapoptotic effects [154].

In clinical practice, PL has been employed in ocular graft-versus-host disease (GvHD) treatment. In a prospective cohort study conducted by Pezzotta et al., the influence of PL eye drops on 61 eyes of 31 patients with ocular GvHD was examined. The treatment resulted in the improvement of ocular symptoms in all studied patients, even in those with worsening GvHD in other organs. After 6 months, all patients experienced improvement in symptoms assessed using the Glaucoma Symptom Scale, TBUT, Oxford lissamine scale classification, and corneal extension grade scale. The reduction in Oxford fluorescein corneal staining was observed in 20 patients (66%). The improvement was still present after 3 years of treatment [155]. The positive effects of PL in ocular GvHD were later shown in subsequent studies [156,157], including in terms of fibrinogen-depleted human platelet lysate (FD hPL) obtained from healthy donors’ pooled blood [158]. Fea et al. reported that, in dry eye related to primary Sjögren syndrome, PL improved OSDI, TBUT, fluorescein score, and Ocular Protection Index values in comparison to artificial tears after 90 days of treatment. Furthermore, using in vivo confocal microscopy, the authors observed a statistically significant increase in basal epithelium cell density, the number of subnasal nerve fibers, and the density of innervation, as well as a decrease in Langerhans cell density, suggesting PL’s ability to regenerate nerves and its anti-inflammatory activity [159]. PL was also found to be effective at treating corneal ulcers [160] and persistent corneal epithelial defects [161].

PL obtained from umbilical cord blood has also been the subject of several studies. In vitro assays on corneal epithelial cells isolated from rats showed that platelet lysate derived from cord blood enhanced their migration and proliferation [162]. The effectiveness of umbilical cord PL in treating eye surface diseases was presented in a multicenter, retrospective, consecutive case study by Samarkanova et al. The authors reported full ocular surface recovery in 25 (78%) and partial in 6 (19%) of 32 eyes with either corneal ulcers or burns. In eyes with ocular GvHD or severe dry eye syndrome, the improvement was noted in 12 (85%) of the studied eyes [163].

## 6. Fourth-Generation Blood Derivatives—Recombinant and Bioengineered Alternatives

The most recent generation of ocular surface biologics represents a shift from blood-derived preparations toward fully engineered or recombinant molecules designed to replicate key tear components under controlled laboratory conditions. These agents, including recombinant human NGF [164], recombinant EGF [165], lacritin analogs [166], and other peptide-based tear substitutes, aim to provide targeted biological activity without reliance on donor material. By acting on specific molecular pathways involved in epithelial repair, neural restoration, and inflammatory regulation, these therapies offer a new level of precision. The clinical success of agents such as the recombinant human nerve growth factor Cenegermin in neurotrophic keratitis [167] underlines the therapeutic potential of their class, while additional recombinant factors remain under active investigation for dry eye disease and epithelial healing disorders.

The topical administration of Cenegermin (recombinant human nerve growth factor, rhNGF) in patients with moderate-to-severe neurotrophic keratopathy resulted in significantly higher rates of complete corneal healing at eight weeks compared to vehicle (65.2% vs. 16.7%; 95% CI 24.0–73.1; *p* < 0.001) [168].

Long-term follow-up of treated patients revealed sustained efficacy over up to 48 months, with low epithelial defect recurrence rates s and improved corneal sensitivity [169].

Similarly, recombinant human epidermal growth factor (rhEGF) has shown promising results in pre-clinical and early-phase human studies: in rabbit corneal wound-healing models, 100 µg/mL of rhEGF administered four times per day accelerated epithelial closure by approximately 45% compared to controls (0.13 vs. 0.09 mm/h) in a re-epithelialization model [170].

In human subjects, a Phase 1 pharmacokinetics and safety study of rhEGF eye drops (at 10–100 µg/mL) demonstrated good tolerability and no systemic exposure concerns [171].

**Figure 4:**
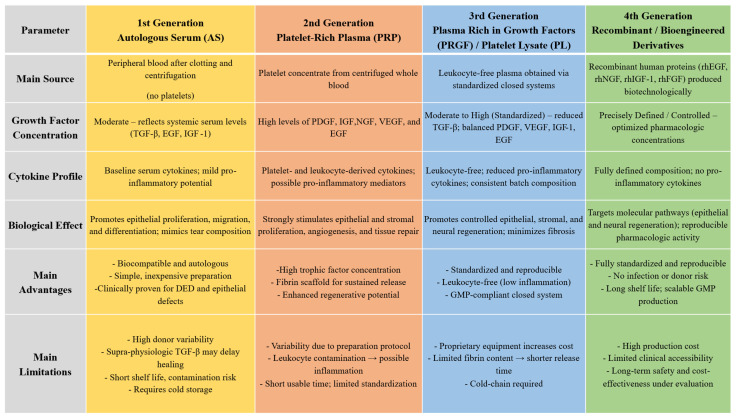
Comparison of four generations of blood derivatives.

The comparison of the main parameters of the four generations of blood-derivatives is presented in Figure 4.

## 7. Finger-Prick Autologous Blood

With the growing interest in blood-derived therapies for ocular surface diseases and the continued development of successive of product generations, attention has also turned toward simpler and more affordable approaches that are based on the abundance of growth factors and bioactive proteins present in blood. The preparation of AS is associated with substantial costs, which further increase with the rising complexity of manufacturing observed across newer generations of blood-derived preparations [172]. In this context, the use of finger-prick autologous blood has emerged as a low-cost, minimally invasive, and accessible alternative. This method involves applying a small droplet of freshly obtained capillary blood, collected via single fingertip puncture, directly onto the ocular surface [173]. The potential advantages of this technique include its low cost, the possibility of higher densities of biologically active molecules, the absence of anticoagulants or storage requirements, and its immediate availability at the point of care. However, the approach also carries notable risks, including ocular infection due to the transfer of skin pathogens, the potential introduction of blood-borne pathogens, and finger tissue damage resulting from repeated punctures [174]. To date, only a limited number of studies have evaluated the clinical efficacy of this method. In the only RCT conducted so far, Hassan et al. investigated the addition of finger-prick autologous blood to conventional DED therapy. The authors reported a statistically significant improvement in OSDI scores and a non-significant trends toward improvement in fluorescein ocular staining scores and TBUT, with no observed benefit regarding Schirmer’s test results [175]. Despite the limited evidence of its efficacy and the constraints of its use, finger-prick autologous blood remains an interesting adjunctive option within the spectrum of biological therapies for ocular surface regeneration, especially in settings where more advanced blood-derived preparations are not readily accessible.

## 8. Blood-Derived Therapies in Clinical Guidelines for Ocular Surface Diseases

Blood-derived preparations in the treatment of ocular surface diseases have been included in both the second and third editions of the Tear Film & Oculsr Surface Society Dry Eye Workshop reports (TFOS DEWS II and TFOS DEWS III).

The TFOS DEWS II report positioned blood-derived preparations as a third-line therapy within its stepwise management algorithm, recommending their use in cases of moderate-to-severe dry eye that are refractory to conventional first- and second-line treatments. The authors highlighted that these preparations mimic the biochemical composition of natural tears, containing growth factors and other bioactive molecules that support ocular surface homeostasis and epithelial healing. However, the report also emphasized several limitations, including lack of standardization in preparation and administration methods, limited patient accessibility, risk of contamination, and the insufficient quality of existing clinical evidence on their efficacy in treating ocular surface diseases [176].

**Figure 5:**
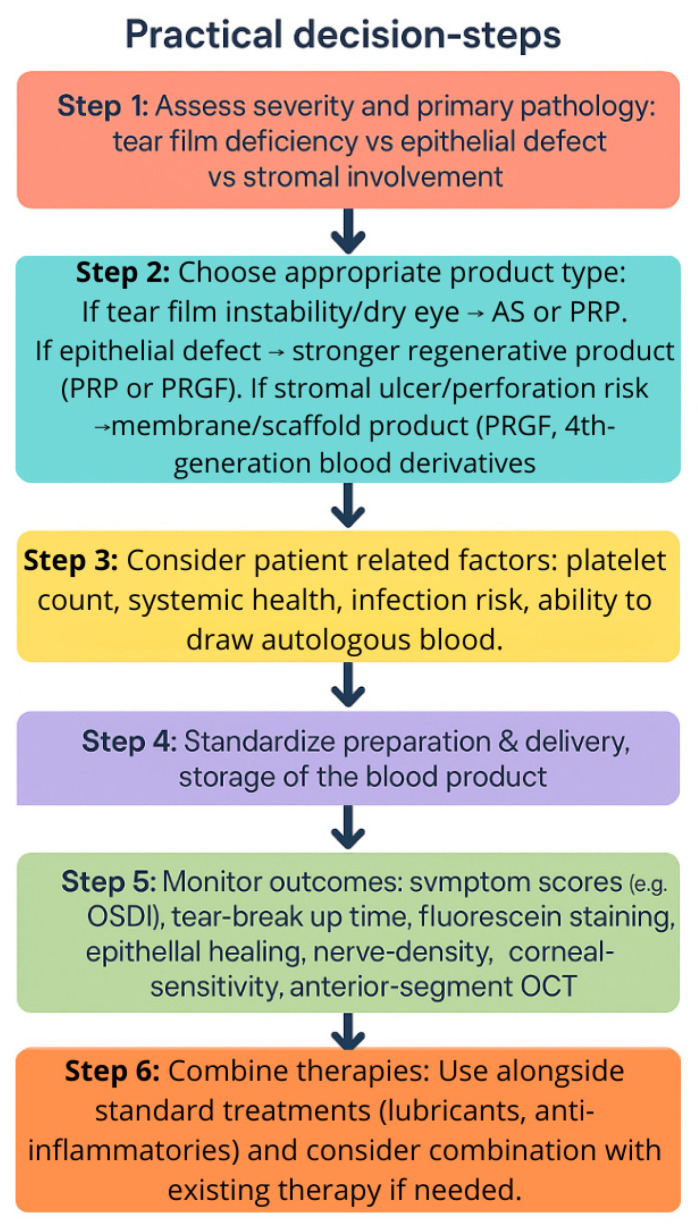
Practical decision tree.

The most recent TFOS DEWS III update incorporated the latest clinical studies and meta-analyses on the use of blood-derived preparations in ocular surface disease management. It acknowledges their beneficial effects on clinical parameters and subjective symptoms of dry eye disease, while at the same time pointing to the need for further RCTs to confirm their efficacy and for greater standardization of preparation and application protocols. Importantly, the new report departs from the rigid hierarchical model of treatment introduced in DEWS II, replacing it with a more flexible, patient-centered approach. Consequently, the use of blood-derived preparations is now viewed as a part of personalized treatment strategy that is tailored to the individual characteristics and underlying mechanisms of DED in each patient [177] (Figure 5).

## 9. Current Limitations and Future Directions

Blood derivatives offer a promising therapeutic option for ocular surface disease.

Autologous serum, allogeneic serum, umbilical cord serum, platelet-rich plasma (PRP), platelets rich in growth factors (PRGFs), and platelet lysate (PL),represent a paradigm shift from conventional symptomatic treatments toward bioactive, patient-tailored therapies capable of stimulating real tissue repair. Our review highlights a clear evolutionary trajectory: from serum-based approaches that mimic tear biochemistry to platelet-enriched formulations offering enhanced regenerative potency through concentrated growth factor release. Among these, PRGF currently stands out as the most refined and standardized preparation, combining efficacy, safety, and reproducibility.

However, despite increasing attention and clinical use of blood-derived products in the regenerative management of ocular surface disease, there are several significant limitations.

Currently, the principal and technical challenge is the lack of standardized preparation and storage protocols—such as various blood collection techniques, different volumes of blood processed, clotting time, number of centrifugations and their parameters (speed, force, duration, and temperature), purification filters, the anticoagulant and/or diluent used, and the activation methods. All these factors influence variability in the composition of the preparation, thus its reproducibility, predictability, and molecular mechanism of action and therapeutic outcomes.

It is essential to acknowledge patient-specific variables—including age, sex, systemic health status, concomitant medications, and nutritional profile—as these factors collectively contribute to inter-batch variability in the composition of autologous preparations. Such variability poses significant challenges to experimental reproducibility, standardization, and the reliable comparison of clinical outcomes across studies.

Furthermore, limited shelf life and requirements for specialized cold-chain storage significantly restrict accessibility and routine clinical application. The lack of universally accepted quality control standards and regulatory frameworks continues to impede large-scale clinical implementation. Moreover, the potential risk of microbial contamination, coupled with insufficient long-term safety data, underscores the necessity for standardized manufacturing protocols and comprehensive clinical validation.

Research on the clinical effectiveness of blood derivatives in ocular surface disease remains restrained by several methodological and practical limitations, which substantially impede the ability to draw firm conclusions or meaningfully compare results between studies. The number of randomized controlled trials is very limited, and most of the available evidence is derived from non-randomized or observational studies, which inherently carry a risk of bias and provide lower-quality evidence [177]. There is considerable heterogeneity across studies, with the age of the patients and underlying disease severity and etiology varying widely, while sample sizes tend to be small and drawn from single-country cohorts, limiting generalizability [53,54,55,56]. Moreover, the sex distribution is markedly imbalanced, with women comprising the vast majority of study participants, which, to some extent reflects the epidemiology of dry eye disease but, on the other hand, restricts the applicability of findings to the general population, and particularly to men [8]. Many trials are also characterized by short follow-up periods, limiting assessment of long-term efficacy and safety [53]. Even in randomized studies, complete masking is challenging, as blood-derived preparations typically have a more viscous consistency and yellowish color, making them visually and tactilely distinct from the clear, less viscous artificial tears commonly used as controls [54]. In several studies, no washout period was implemented before initiating treatment, making it difficult to distinguish the therapeutic effect of the tested preparation from the residual effect of previously used agents [48,54]. Additional confounders include the frequent concomitant use of systemic medications, which may influence ocular surface status [66,88], and the elapsed time between blood collection and product processing, which differs significantly between blood-derivative types, particularly allogeneic and autologous preparations, and may influence the final concentration of bioactive molecules [89]. Finally, many trails omit assessments of quality of life and cost-effectiveness, despite their relevance for evaluating real-world clinical utility and informing health-related and economic decisions [56]. Collectively, these factors complicate direct comparison between studies and limit the strength of the evidence base supporting the clinical use of blood-derived therapies.

To address these limitations, future research should move beyond general optimization and focus on developing more standardized, evidence-based preparation methods and technologically advanced strategies that ensure reproducibility and consistent bioactivity.

An important future direction in the field of blood-derived therapeutics lies in the development of personalized preparations integrating proteomic and metabolomic analysis, that provides a molecular understanding of inter-patient variability, enabling the design of personalized blood-derived products optimized for specific regenerative needs.

Moreover, recent progress in biomaterials and bioengineering opens the door to developing cell-free, off-the-shelf growth factor cocktails that replicate the therapeutic effects of autologous derivatives without patient blood donation. Finally, combining blood-derived therapies with novel biomaterials or drug-delivery systems may further extend their therapeutic potential by improving stability and targeted delivery.

In conclusion, blood derivatives hold exceptional promise as next-generation regenerative therapies for ocular surface diseases. Their future success will depend on rigorous scientific standardization, technological innovation, and a shift toward personalized precision medicine.

## 10. Materials and Methods

A comprehensive review of the literature was conducted to identify studies investigating the use of evaluate blood derivatives and their role in the treatment of ocular surface diseases. The search for relevant studies in the English language, published up to September 2025, was performed using electronic databases including PubMed/MEDLINE, Embase, Web of Science, Scopus, and the Cochrane Library. The following terms and their combinations were used: “platelet-rich plasma” OR “PRP” OR “autologous serum” OR “AS” OR “plasma rich in growth factors” OR “PRGF” OR “blood hemoderivatives” OR “umbilical cord” OR “platelet lysate” AND “ocular surface” OR “ocular surface disease”.

Additionally, reference lists of relevant articles and review papers were manually screened to identify further eligible studies. The studies were included if a full-text article in English was available. Conference abstracts, case reports, and studies with insufficient methodological detail were excluded.

The selection of the studies was carried out based on their eligibility, relevance to the subject, focus on clinical outcomes, mechanisms, and the safety profiles of blood-derived therapies in ocular surface diseases. The reference lists of included articles were reviewed for any additional applicable studies.

The graphics in this paper were created with the assistance of generative artificial intelligence.

## Figures and Tables

**Table 2:** Randomized controlled trials (RCTs) on topical use of allogeneic serum in treating ocular surface disorders.

Reference	Year	Study Design	Study Group (*n*)	Control Group (*n*)	Treated Condition	Intervention	Comparator/Control	Preparation	Dosage (Times/Day)	Duration	Outcomes Measured	Outcomes Significantly Improved Compared to Control
van der Meer et al. [96]	2021	Crossover RCT	15	15	severe DED	AlS	50% AS	AlS: whole blood from never-transfused male repeat donors with blood group AB → clot for 6–24 h at room temperature → centrifugation with an Accumulated Centrifugal Effect (ACE) of 9 × 10^7^→ extraction of serum → centrifugation at 6580 g for 7 min → dilution to 50% with saline AS: whole blood → clot for 6–24 h at room temperature → centrifugation with an Accumulated Centrifugal Effect (ACE) of 9 × 10^7^ → extraction of serum → centrifugation at 6580× *g* for 7 min → dilution to 50% with saline	6	1 month	OSDI, TBUT, BCVA, OSS, ST	No significant differences were found
Rodríguez Calvo-de-Mora et al. [89]	2021	Three-arm RCT	21 (AS), 21 (AlS), 21 (UCS)	N/A	severe DED	AS, AlS, UCS	N/A	UCS: Umbilical cord blood → centrifugation → dilution to 20% with solution of Sterile Irrigating Solution, BSS^®^ AS: whole blood → centrifugation → dilution to 20% with solution of Sterile Irrigating Solution, BSS^®^ AlS: whole blood form AB-blood donors → centrifugation → dilution to 20% with solution of Sterile Irrigating Solution, BSS^®^	5	3 months	ST, TBUT, OSS	No significant differences were found

## Data Availability

The original contributions presented in this study are included in the article. Further inquiries can be directed to the corresponding author.
